# Visual electrophysiology and “the potential of the potentials”

**DOI:** 10.1038/s41433-023-02491-2

**Published:** 2023-03-16

**Authors:** Omar A. Mahroo

**Affiliations:** 1 Institute of Ophthalmology, University College London, 11-43 Bath Street, London, UK; 2 Retinal and Genetics Services, Moorfields Eye Hospital, 162 City Road, London, UK; 3 Section of Ophthalmology and Department of Twin Research and Genetic Epidemiology, King’s College London, St Thomas’ Hospital Campus, Westminster Bridge Road, London, UK; 4 Physiology, Development and Neuroscience, University of Cambridge, Downing Street, Cambridge, UK; 5 Department of Translational Ophthalmology, Wills Eye Hospital, Philadelphia, PA USA

**Keywords:** Retinal diseases, Retina

## Abstract

Visual electrophysiology affords direct, quantitative, objective assessment of visual pathway function at different levels, and thus yields information complementary to, and not necessarily obtainable from, imaging or psychophysical testing. The tests available, and their indications, have evolved, with many advances, both in technology and in our understanding of the neural basis of the waveforms, now facilitating more precise evaluation of physiology and pathophysiology. After summarising the visual pathway and current standard clinical testing methods, this review discusses, non-exhaustively, several developments, focusing particularly on human electroretinogram recordings. These include new devices (portable, non-mydiatric, multimodal), novel testing protocols (including those aiming to separate rod-driven and cone-driven responses, and to monitor retinal adaptation), and developments in methods of analysis, including use of modelling and machine learning. It is likely that several tests will become more accessible and useful in both clinical and research settings. In future, these methods will further aid our understanding of common and rare eye disease, will help in assessing novel therapies, and will potentially yield information relevant to neurological and neuro-psychiatric conditions.

## 摘要 摘要

视觉电生理学能直接、定量、客观地评估不同水平的视觉通路功能, 因此可获得从影像学或心理物理测试中不一定能获得的互补信息。随着技术进步和对波形神经基础的理解的加深, 现有的测试及其指南的发展有助于更精确地评估视觉同路生理学和病理生理学的改变。本文总结了视觉通路和当前的标准临床测试方法, 不完全地讨论了几个设备, 尤其关注人类视网膜电图记录。其中包括新的设备(便携式、免散瞳、多模式)、新的测试指南(包括旨在分离视杆驱动和视锥驱动反应, 和监测视网膜适应的指南), 以及建模和机器学习的使用等分析方法的发展。在临床和研究中, 有几种测试可能便于获取并且具有实用性。在未来, 这些方法将进一步帮助我们了解眼部的常见和罕见病, 有助于评估新的治疗方法, 并可能提供与神经和神经精神疾病相关的信息。

## Introduction

Transformational advances have occurred in recent decades in multimodal imaging of the retina and visual pathway. Such techniques, however, largely convey information on anatomical structure. Electrophysiology permits direct, objective and quantitative assessment of function, both at the level of the visual cortex (visual evoked potentials, VEPs) and at the level of the retina (electroretinogram, ERG). This review will briefly summarise the visual pathway, list current standard electrophysiological testing protocols, and then describe some recent advances and anticipated future developments, with particular focus on the ERG.

## The neuronal pathway of visual signals

After refraction and transmission by the optical media, light hits the retina, isomerising photopigment and initiating the phototransduction cascade in the outer segments of the rod and cone photoreceptors. This results in the shut-off of a cationic current (that was flowing into the outer segments in the dark), leading to hyperpolarisation of the photoreceptors (the membrane potential becoming more negative). This in turn leads to reduction in glutamate release at the synapse between photoreceptors and bipolar cells. ON bipolar cells depolarise (membrane potential becomes more positive) in response to this, whilst OFF bipolar cells hyperpolarise. Bipolar cells synapse with ganglion cells, whose axons form the optic nerve. Other neuronal cell types in the retina, horizontal cells and amacrine cells, modify transmission at these synapses, subserving lateral interactions between photoreceptors and bipolar cells respectively, such that the information transmitted in the ganglion cell axons represent highly processed features of the visual environment.

The optic nerves (formed from the axons of the retinal ganglion cells) from each eye meet at the optic chiasm: the fibres originating from the nasal retina of each eye decussate to join the contralateral optic tract, whilst those originating in the temporal retina continue in the ipsilateral optic tract. The ganglion cell axons synapse with neurons in the lateral geniculate nucleus, with the latter neurons then projecting in the optic radiations to the primary visual cortex, located in the occipital lobes. Thus, the right visual cortex receives input from the temporal retina of the right eye and the nasal retina of the left eye (both responding to stimuli in the left visual field), whilst the left visual cortex receives input from the temporal retina of the left eye and the nasal retina of the right eye (thus responding to stimuli in right visual space).

## Visual electrophysiology tests

### Full-field electroretinogram

**Fig. 1:**
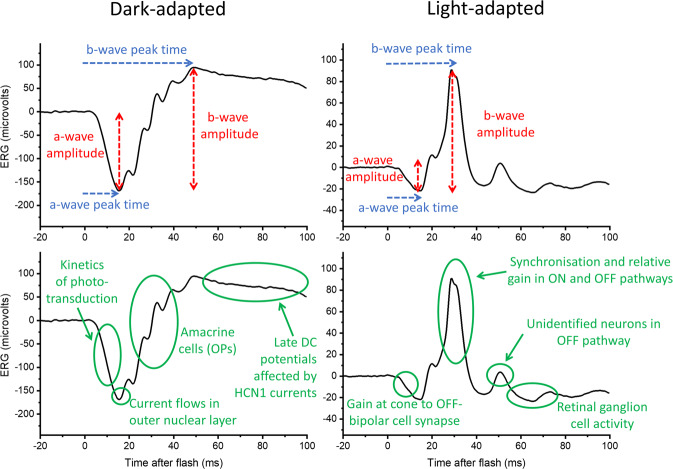
ERG responses to white flashes delivered in the dark-adapted and light-adapted state. Traces show ERG responses (averaged from several flash presentation) recorded from a healthy individual to strong flashes (10 photopic cd s m^−2^, corresponding to the DA10 of the ISCEV standard) delivered in the dark following dark adaptation (lefthand panels) and to standard flashes (3 photopic cd s m^−2^, corresponding to the LA3 of the ISCEV standard) delivered on a standard white background (30 photopic cd m^−2^) in the light-adapted state (righthand panels). Labels in the upper panels highlight the quantitative parameters usually reported (a-wave and b-wave amplitudes and peak times); the oscillatory potentials (OPs) on the rising limb of the b-wave may be qualitatively evaluated or quantified with additional filtering. The lower panels highlight several components in the waveforms and the underlying retinal processes or neuronal origins that could potentially be interrogated quantitatively with more sophisticated analyses (including mathematical modelling or machine learning techniques).

The electroretinogram (ERG) represents the summed electrical response of the retina to light stimuli. The potential difference is measured between a corneal electrode and a reference skin electrode (the latter usually placed on the temple), with a ground electrode commonly placed on the forehead. The full-field ERG [1] is elicited by flashes that stimulate the whole of the retina. This typically consists of a negative deflection, the a-wave, which largely reflects the hyperpolarisation of the photoreceptors, followed by a positive deflection, the b-wave, that largely reflects the depolarisation of the ON bipolar cells. The amplitudes and peak times of these components are conventionally measured, as shown in the upper panels of Fig. 1. (The lower panels of Fig. 1 highlight additional features of the waveforms, which will be discussed in a later section.)

Selective attenuation of the b-wave suggests a locus of dysfunction that occurs after phototransduction (for example owing to impairment of bipolar cell function or reduced synaptic transmission between photoreceptors and bipolar cells) [2, 3], whilst attenuation of the a-wave indicates impairment of phototransduction. Stimuli are conventionally delivered in the dark-adapted state, to assess rod system function, and in the light-adapted state, to assess cone system function. As well as flash stimuli in the light-adapted state, a flicker stimulus (conventionally at 30 Hz, which cannot be resolved temporally by the rod system) is also used to evaluate cone system function [1]. With conventional full-field ERG stimuli, therefore, retina-wide dysfunction can be detected, and impairments can be localised to the rod system or cone system, and to the outer retina (photoreceptor outer segments) or inner retina (impairments that occur after phototransduction, affecting the photoreceptor bipolar cell synapse or bipolar cell signals).

### Pattern electroretinogram

The pattern ERG [4] is elicited by a checkerboard stimulus (with alternating black and white squares reversing a few times a second) that stimulates the central 15 degrees of retina, thus testing macular function. A positive component (peaking at around 50 ms, hence termed the P50) is seen followed by a negative component (the N95, with a trough around 95 ms). Abnormalities of the pattern ERG can arise from dysfunction of the macula, including the macular cone photoreceptors, and also the macular retinal ganglion cells (in the latter case, the N95 is more severely affected than the P50).

### Multifocal electroretinogram

The multifocal ERG [5] also gives information relating to more localised areas of retina than the full-field ERG. Here, an area of retina of around 40 or 50 degrees is stimulated by an array of light and dark hexagons, each of which reverses in luminance according to a pseudorandom sequence. The resulting ERG recording is then mathematically correlated with each hexagon’s sequence of illumination to yield waveforms (typically a negative deflection, followed by a positive, followed by a second negative, deflection, labelled N1, P1, and N2 respectively) corresponding to each specific area of retina (the area stimulated by the relevant hexagon).

### Electro-oculogram

The electro-oculogram (EOG) [6] measures the standing potential of the eye, relating to a potential across the retinal pigment epithelium (RPE). Skin electrodes are placed at the inner and outer canthus of each eye, and patients make horizontal saccades between two targets. As the eyes move, the potential across the RPE reverses relative to the electrodes, resulting in deflections seen in the recording. In the dark, the amplitudes of these deflections fall to a minimum (the “dark trough”), and in the light, they rise to a peak (the “light peak”) over the course of several minutes. The light peak:dark trough ratio is selectively reduced in certain conditions (whilst the ERG is normal), typically in Best disease.

### Visual evoked potentials

Visual evoked potentials (VEPs) [7] are recorded from electrodes placed on the scalp and overlying the visual cortex. These can be evoked by flash stimuli or patterned stimuli (the latter are in a checkerboard configuration of light and dark squares, similar to the stimulus for the pattern ERG). Abnormalities can arise from impairments anywhere in the visual pathway, and so VEPs are usually best interpreted in the context of ERG findings, for example in distinguishing between a retinopathy/maculopathy or optic neuropathy. Pattern reversal VEPs (in which the black and white squares reverse) show least variability between individuals. Pattern onset VEPs can give objective estimates of visual acuity (by evaluating the smallest check sizes that appear to be resolved at the level of the visual cortex) [8, 9], helpful in cases of suspected non-organic visual loss. Flash VEPs can be particularly useful in cases of poorer cooperation and fixation, or when optical factors might degrade a patterned image.

VEPs can aid in evaluating optic nerve disease and multi-channel VEPs can help detect intracranial misrouting associated with albinism. In albinism, the majority of retinal ganglion cell axons cross at the chiasm rather than solely those from the nasal retina. Thus, whilst in healthy individuals a monocular stimulus will produce similar VEP responses over both hemispheres, in albinism, a monocular stimulus produces a larger response over the contralateral hemisphere.

## ISCEV standard protocols

Visual electrophysiology waveforms are dependent upon stimulus and recording parameters, as well as physiological or pathological changes in the visual pathway. The International Society for Clinical Electrophysiology of Vision (ISCEV) defines standard protocols, which enables some uniformity across laboratories and in publications. The standards are revised periodically. Currently there are standards for the full-field ERG, the pattern ERG, multifocal ERG, EOG and VEP [1, 4–7]. Table 1 lists the standard protocols along with the year of most recent update, and summarises the relevant stimuli. ISCEV also publishes extended protocols [9–16], which will be discussed later.

**Table 1:** International Society of Clinical Electrophysiology of Vision (ISCEV) standard protocols with reference to latest updated versions at the current time (Jan 2023).

Test	Latest update	Authors	Summary Comments
Full-field ERG	2022	Robson et al.	White full-field stimuli delivered in the dark-adapted (DA) state (following 20 min dark adaptation) and in the light-adapted (LA) state (in the presence of a 30 cd m^−2^ white background, following 10 min adaptation to this background if dark-adapted responses have been recorded first). DA responses are to 0.01, 3 and 10 cd s m^−2^ flashes. LA responses are to 30 Hz flicker and to flashes (both 3 cd s m^−2^). Pupils should be dilated. If non-mydriatic responses are recorded, stimulus strength should be adjusted to give equivalent retinal illuminance. The document also includes a non-standard abbreviated protocol that can be helpful for example with very young patients.
Multi-focal ERG	2021	Hoffmann et al.	Stimulus display consists of 61 or 103 scaled hexagons (recommended total recording time of 4 or 8 min respectively), with frame rate between 60 and 75 Hz. Luminance of elements in the light state should be 100 cd m^−2^ or more. Pupils are dilated. Fixation is important.
Pattern ERG	2012	Bach et al.	Stimulus is contrast-reversing checkerboard pattern (luminance of white areas should be 80 cd m^−2^ or more), with reversal rate of 4.0 reversals per second. Pupils are not dilated; fixation and optimal refraction are important. Stimulus field should be 15 degrees in width and height. Check size is 0.8 degrees.
Electro-oculogram	2017	Constable et al.	Stimuli presented within a uniform field (integrating sphere). Pupils are dilated. Electrodes placed at canthi. Patient makes saccades between two red fixation lights (15 degrees left and right of centre) that alternate every second for 10 s in every minute, in the dark for 15 min, and then in a white light background (100 cd m^−2^) for 15 min. The light peak to dark trough ratio is calculated.
Visual evoked potentials	2016	Odom et al.	VEPs are recorded to pattern (checkerboard) stimuli (subtending 15 degrees; mean luminance 50 cd m^−2^) and to flash stimuli, presented monocularly. Pattern-reversal VEPs (2 reversals per second), and pattern onset/offset VEPs are recorded to checkerboard stimuli with large (1 degree) and small (0.25 degree) checks. Flash VEPs are elicited by a flash (3 cd m^−2^ s) subtending 20 degrees or more of visual field. A single midline occipital active electrode is used (multi-channel recording is not part of the basic standard, but needed to assess chiasmal and post-chiasmal pathway dysfunction and chiasmal misrouting). Pupils not dilated.

Stimulus strengths are in photopic units. The reader is referred to the relevant documents for full details.

## Some current and future developments

There are numerous recent and anticipated developments in the field of visual electrophysiology. In 2021, a special section in this journal featured several relevant articles, each focusing on a particular aspect, summarising current knowledge, providing new insights and future directions [3, 17–24]. For the remainder of the current article, some advances, particularly relating to human ERGs, will be highlighted, categorised broadly into new devices, new stimulus protocols, and novel analyses. These are also summarised in Table 2.

**Table 2:** Examples of developments in visual electrophysiology (list not exhaustive), some of which are described in more detail in the text.

**Developments**	**Examples**
Advances in devices	•Portable/handheld equipment •Real-time pupil monitoring and adjusting of stimulus strength to deliver desired retinal illuminance, allowing non-mydriatic recordings •Multimodal platforms combining psychophysical and electrophysiological testing, or combining high-resolution fundus imaging (including OCT) with electrophysiology
Stimulus protocols	•ISCEV extended protocols •Protocols to separate dark-adapted rod and cone responses or to selectively modulate specific photoreceptor classes (silent substitution) •Dynamic tracking of retinal light and dark adaptation using electrophysiology
Novel analyses	•Waveform transformations •Fitting of mechanistic models to derive parameters of phototransduction or outer retinal current flows in the living human eye •Artificial intelligence/machine learning algorithms •Genetic analyses (association of electrophysiology parameters with common and rare genetic variants)

### Advances in devices

For decades, xenon flash guns and incandescent lamps were used to provide flash and background stimuli. Developments in LED technology allowed more energy efficient devices and more precise control and stability of stimuli. Over 20 years ago, one hurdle in the investigation of kinetics of recovery of human cone photoreceptor circulating current at sub-second time scales following very intense steady bleaching exposures was the time taken to extinguish (or for the subject to move away from) the steady bleaching background [25]. With the availability of ultrabright LEDs, bright bleaching backgrounds could be extinguished rapidly, and bright xenon flashes could be delivered within tens of milliseconds following background extinction, helping establish that cone circulating current recovers extremely rapidly: following a bleaching exposure estimated to bleach around 90% of the cone photopigment, cone circulating current, as estimated by the bright-flash ERG appeared to recover fully within 100 ms (and substantially within 20 ms) [26]. For rods, following a similar large bleach, recovery of circulating current takes at least 20 min; [27] thus, recovery of current in cones was shown to occur over 10,000 times faster [26].

#### Portable nonmydriatic devices

**Fig. 2:**
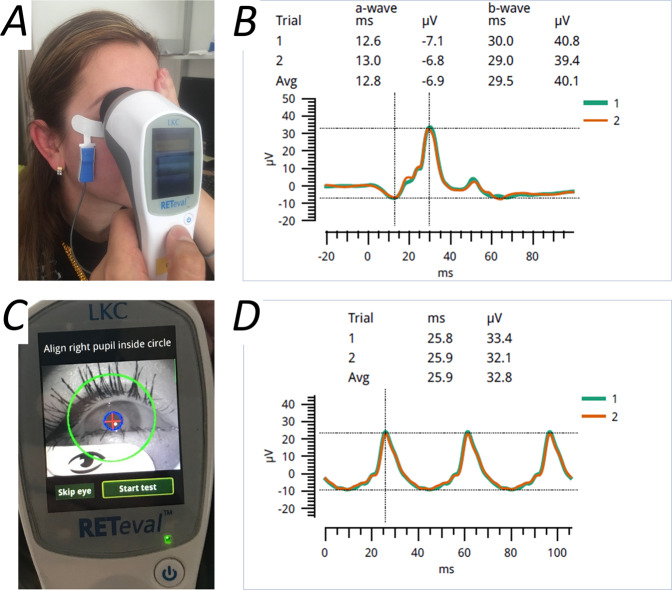
A portable nonmydriatic stimulus and recording system (RETeval, LKC Technologies). **A** Demonstration of positioning of device over the eye from which recordings are being taken. The electrodes being used are the “Sensor Strip” skin electrodes (LKC Technologies), but the device can also be used in conjunction with other electrode types. **B** Example ERG responses recorded with this technique in response to a stimulus equivalent to the standard light-adapted flash (each trace is the average of approximately 30 flash presentations; the green and orange traces are from two successive series of flashes). **C** View of device screen prior to initiation of nonmydriatic recordings. The device has an inbuilt video camera, so the subject’s eye is visible. The device also detects the pupil (highlighted by blue circle) and will adjust stimulus strength to deliver the retinal illuminance equivalent to that delivered by standard stimuli through a dilated pupil. **D** Responses to a stimulus equivalent to the standard light-adapted 30 Hz flicker.

Whilst handheld stimulators have been available for many years, more sophisticated portable devices have been developed more recently. The RETeval device (LKC Technologies) can deliver near full-field stimuli equivalent to the ISCEV standard, and can be used with a variety of electrodes, including “Sensor Strip” skin electrodes, which are placed on the skin below the lower lid margin and which incorporate a recording, reference and ground electrode within the same strip. The device includes a camera, so the subject’s eye can be viewed (and eye closure noted) by the operator. The device also measures pupil diameter and can adjust stimulus strength accordingly (to match the retinal illuminance delivered by standard stimuli through a dilated pupil), which allows use with natural pupils, removing the need for pharmacological mydriasis. Figure 2 illustrates the device and example waveforms. Many publications have emerged reporting its use in diverse conditions including diabetic retinopathy, a range of inherited retinal diseases, birdshot chorioretinopathy, glaucoma, idiopathic intracranial hypertension, and others [28–47].

#### Multimodal devices

Another important development in device technology is multimodality. Both historically and currently, separate pieces of equipment are used for psychophysical testing (including perimetry), retinal imaging and electrophysiology. More recently, some manufacturers have combined psychophysical testing capability and electrophysiology within the same device, for example dark adaptometry, full-field stimulus testing (FST), combined with electrophysiology (Diagnosys LLC) [48, 49]. Fundus cameras have also been used in conjunction with ERG to reliably localise the area of stimulation [50, 51]. Very recently, a system has been developed incorporating both optical coherence tomography (OCT) and ERG recording, aiming to stimulate very small areas of retina with OCT guidance, and record responses (Nanoscope Technologies LLC) [52].

### Newer stimulus protocols

#### ISCEV extended protocols

In addition to the well-established ISCEV standard protocols, several extended protocols [9–16], endorsed by ISCEV, have been developed to probe, in more detail, aspects of visual function. These are summarised in Table 3. Some have been incorporated in portable devices, with findings reported in several studies [3, 31, 33, 35, 36, 39]. An ISCEV guide has been published reviewing the range of standard and extended protocols [53].

**Table 3:** ISCEV extended protocols with summary notes and year of latest update. All stimulus strengths are in photopic units unless otherwise specified.

Protocol	Latest update	Authors	Summary Comments
VEP estimation of visual acuity	2021	Hamilton et al.	Guidelines for recording pattern VEPs to range of check sizes to establish spatial frequency limit and relating this to visual acuity. Helpful to estimate visual acuity when conventional psychophysical testing not possible or reliable.
Derivation and analysis of strong flash rod-isolated ERG a-wave	2020	Brigell et al.	ERGs recorded to a strong white flash (30 cd s m^−2^) under dark adaptation and additionally following light adaptation in standard background (30 cd m^−2^). Estimated rod-driven ERG a-wave derived by subtracting light-adapted (cone-driven) response from the dark-adapted (rod and cone-driven ERG). Potential value in characterisation of diseases selectively affecting rod phototransduction or numbers of rod photoreceptors.
S-cone ERG	2020	Perlman et al.	ERGs recorded in response to a blue flash (450–470 nm) delivered on a longer wavelength (yellow, amber or orange) background (background peak wavelength between 570–620 nm, luminance 300 cd m^−2^). Helpful in distinguishing between achromatopsia and blue cone monochromacy, and in confirming diagnosis of enhanced S-cone syndrome.
Stimulus response series, light-adapted ERG	2019	McCulloch et al.	Light-adapted ERGs recorded to 9 flash stimuli (0.03 to 300 cd s m^−2^) in presence of standard background (30 cd m^−2^) with guidance on fitting stimulus-response relation. Shorter protocol also provided. Enables more comprehensive characterisation of cone system function than standard light-adapted stimuli, including characterisation of “photopic hill” phenomenon (b-wave amplitude increasing with stimulus strength to peak and then falling to non-zero plateau).
Stimulus response series, dark-adapted b-wave	2019	Johnson et al.	Dark-adapted ERGs recorded in response to large range of flash strengths (typically from −3.5 to 0.5 log cd s m^−2^) with guidance on fitting of stimulus-response relation. Provides more comprehensive characterisation of rod system function than standard dark-adapted stimuli.
Photopic negative response (PhNR)	2018	Frishman et al.	The PhNR is a negative waveform component following the b-wave and arises from retinal ganglion cells (RGCs). The recommended stimulus is a red (630–660 nm) LED flash (1.0–2.5 cd s m^−2^) on a blue (450–485 nm) LED background (100 scotopic cd m^−2^). Potential use in evaluating RGC function (in glaucoma and other optic neuropathies).
Dark-adapted red flash	2018	Thompson et al.	Dark-adapted ERG to dim red flash (0.3 cd s m^−2^), shows an early (30–50 ms) positive cone-driven peak followed by a later (~100 ms) larger rod-driven peak. Allows assessment of dark-adapted cone system, helpful in evaluating cone system dysfunction (particularly retinopathy associated with variants in *RGS9* or *R9AP*), and also in determining origin (rod or cone) of residual standard dark-adapted ERGs.
On-Off ERG	2018	Sustar et al.	ERGs recorded to long duration (150 or 200 ms) stimuli. Stimuli are white (150–350 cd m^−2^) on a white background (30 cd m^−2^), or orange (620 nm) stimuli on a green (560 nm) background. The long stimulus allows separate evaluation of response to stimulus onset and offset. Helpful in distinguishing conditions selectively impairing ON bipolar signals (e.g., complete congenital stationary night blindness and melanoma-associated-retinopathy) from those impairing both ON and OFF signals (incomplete congenital stationary night blindness and other disorders).

The On-Off ERG entails the delivery of long duration (>100 ms) stimuli so that retinal responses to onset and offset of stimulation can be separately evaluated. This can be helpful in evaluating conditions in which ON bipolar cell signals are selectively attenuated (including complete congenital stationary night blindness and melanoma-associated retinopathy) [16]. Delivery of dim red flashes in the dark-adapted state can yield assessment of the dark-adapted cone system [15]. The photopic negative response is thought to be best elicited by red flashes delivered on a blue background [14]. It derives from retinal ganglion cells, and has been shown to be attenuated in optic neuropathies, including glaucoma. Stimulus response series have been published for the dark-adapted and light-adapted ERGs [12, 13], where ERGs are recorded in response to a wider range of flash strengths than in the standard for full-field ERGs. Selective S-cone ERGs (elicited by blue flashes delivered on a longer wavelength background) can help confirm the diagnosis of enhanced S-cone syndrome (typically related to bi-allelic variants in *NR2E3*) and demonstrate S-cone preservation in blue cone monochromacy (helping distinguish it electrophysiologically from achromatopsia) [11]. A published protocol also exists to derive the dark-adapted rod-driven a-wave response to strong flashes (by subtraction of the cone-driven component yielded by the response to the same flash delivered on the standard ISCEV light-adapting background) [10]. Recently, a protocol has been published for estimation of visual acuity from the pattern VEP, using different check sizes to establish the spatial frequency limit [9].

#### Parsing dark-adapted rod and cone responses

**Fig. 3:**
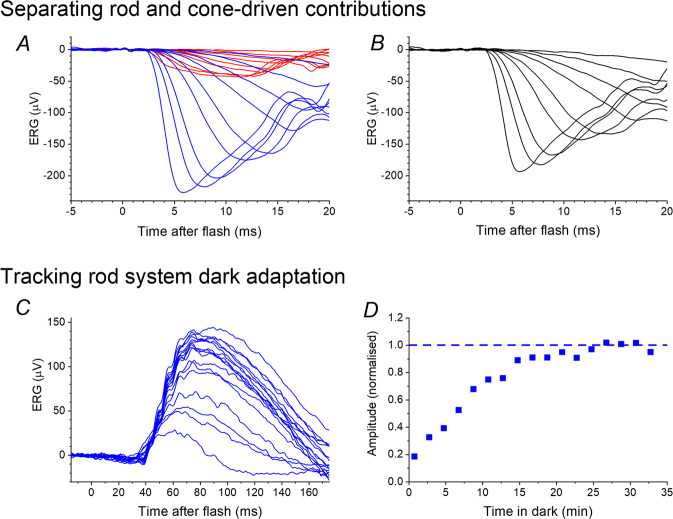
Examples of non-standard stimulus protocols to interrogate specific aspects of retinal function. **A**, **B** Upper panels show a method to separate rod and cone-driven contributions to dark-adapted flash responses. **A** Blue traces show ERG responses to blue flashes of a range of strengths delivered in the dark-adapted state (each trace averaged from multiple flash presentations). These responses contain contributions from rod and cone systems. The red traces show responses to the same flashes, but delivered on a blue background. The background strength was 2.9 photopic cd m^−2^ and 34 scotopic cd m^−2^. Delivered through a dilated pupil, the background illuminance (approximately 1500 to 1700 scotopic trolands) is likely to be sufficient to saturate the rods (which are thought to be largely saturated by backgrounds in excess of 1000 scotopic trolands), but minimally adapt the cones. Thus, the flash responses recorded are cone-driven with no rod-driven components. **B** Traces plotted are the result of mathematical subtraction of the red traces from the blue traces in panel **A**. These traces represent the estimated isolated rod-driven component (with the a-waves largely reflecting current flows in the rod photoreceptors). **C**, **D** Lower panels show an ERG method of tracking recovery of rod system sensitivity in the dark following a bright light exposure. **C** ERG responses to dim flashes of fixed strength (0.02 scotopic cd m^−2^) delivered at different times in the dark after steady state exposure to a standard white background (30 photopic cd m^−2^). The smaller amplitude responses were recorded at earlier times following extinction of the background, whilst the larger amplitude responses were recorded at later times. **D** Amplitudes of responses in **C** plotted as a function of post-bleach time. Amplitudes have been normalised to the estimated final dark-adapted level (denoted by the horizontal dashed line).

Separating rod and cone contributions to responses to strong flashes delivered in the dark can pose a particular challenge, since both rods and cones are stimulated. The extended protocol mentioned above [10] specifies delivery of the same flash in the presence of the standard ISCEV white background to estimate the cone-driven contribution in the dark. However, the standard white background is likely to significantly light adapt the cone system, so the response elicited by a flash delivered on this background is likely to differ somewhat from the cone-driven response to a flash delivered in the dark. Alternative methods take advantage of the different spectral sensitivities of rods and cones: rods are maximally sensitive to shorter (bluer) wavelengths than the majority of cones (which are L- and M-cones, whilst S-cones are relatively sparse). Thus, a blue background can be applied which saturates the rods, but minimally light-adapts the majority of the cones. Responses recorded to flashes delivered on this background will be cone-driven, with no rod-driven component. These responses will more closely match the dark-adapted cone system response than those recorded on the standard white background. If these responses (recorded on the dim blue rod-saturating background) are subtracted from responses to the same flashes delivered in the dark, the result provides an estimate of the rod-driven response (Fig. 3, upper panels). Several studies have used this method [25, 26, 54–58], including a recent investigation into ERG associations of a common myopia-associated variant [58]. In that study, the technique revealed an association specifically with cone-driven, but not rod-driven, responses.

Other methods of separating rod and cone contributions exist, including a double flash protocol whereby flashes are delivered at early times (around 1 second) following a very strong flash [54, 59]. At this time, rods will still be in saturation, and hence the response will be cone-driven. It is also possible to separate photoreceptor classes at particular levels of adaptation (rods and cones, and even L and M cones, which have considerable overlap in wavelength sensitivity) using “silent substitution” methods whereby the spectral composition of stimuli is manipulated such that certain photoreceptor classes (for example L or M cones) can be selectively modulated whilst maintaining the same stimulation of the other photoreceptors. This has been used psychophysically and also in electrophysiology [60–62].

#### Tracking retinal adaptation with the ERG

Conventionally, ERGs are recorded in a steady (either dark-adapted or light-adapted) state. It is possible, however, to use the ERG to dynamically track adaptation to different backgrounds. Whilst dark adaptation has mainly been measured psychophysically, an ERG-based method allows objective and quantitative tracking of recovery of rod and cone sensitivity at the level of the retina. Typically, following a bleaching exposure (steady exposure to bright illumination or to a series of strong flashes), stimuli of a fixed strength are delivered repeatedly in the dark. The ERG elicited by such stimuli is initially of low amplitude, but the amplitude recovers with time in the dark. These studies have given insights into kinetics of rod and cone photopigment regeneration and recovery of photoreceptor outer segment current [27, 56, 57, 63–67]. Figure 3 (lower panels) shows an ERG method of using dim flashes to track rod system recovery in the dark, variations of which have been used in several investigations [63, 65, 66, 68].

Such methods also helped demonstrate the physiological basis of the phenomenon of transient smartphone “blindness” [68]. In these cases, patients reported recurrent monocular nyctalopia, initially eluding medical explanation. Following careful history-taking, the reduced visual sensitivity occurred following smartphone viewing whilst lying in bed on one side (such that one eye was inadvertently covered by the pillow; this eye became dark adapted, whilst the uncovered eye light adapted). By recording ERGs to dim flashes delivered to both eyes at different times following monocular smartphone viewing, it was shown that the phenomenon was simply one of retinal adaptation and demonstrable at the level of rod-driven bipolar cell responses [68]. This ERG method of tracking recovery of rod or cone sensitivity may have utility in conditions in which dark adaptation is selectively affected (including vitamin A deficiency, Sorsby fundus dystrophy, and age-related macular degeneration). Use of the ERG in tracking rod and cone dark adaptation has recently been extensively reviewed, along with discussion of potential applications [69].

### Novel analyses

Figure 1 shows standard dark-adapted and light-adapted full-field flash ERG responses. The upper panels, as mentioned earlier, show the parameters that are conventionally measured and reported. The lower panels highlight multiple components in the waveform and potential inferences that could be made, with more advanced quantitative analyses, regarding physiological and pathophysiological processes in specific retinal neuronal groups. Several papers have used more sophisticated analyses, such as discrete wavelet transforms [70–72], applied for example to separately evaluate ON and OFF components in responses [70]. Frequently, novel analyses are used in conjunction with specifically designed protocols, hence many of these developments have occurred simultaneously.

#### Mathematical models of phototransduction and outer retinal current flows

A mechanistic model, taking into account the likely kinetics of each of the activation stages of phototransduction, was formulated over 30 years ago by Lamb and Pugh [73]. This was shown to provide a remarkably close fit, using a single set of parameters, to changes in the photoreceptor outer segment current in response to a range of flash strengths. This model was also shown to provide a reasonable fit to the leading edge of the ERG a-wave, and was fitted to both rod and cone-driven a-waves in numerous studies [25, 54, 57, 66, 74–81].

However, limitations to this approach have been identified [79, 82]. The model did not provide a close fit to ERG responses to very strong flashes, and so a capacitive time constant was incorporated to improve the fit [77]. Also, the cone-driven a-wave has been shown to contain significant post-receptoral (OFF bipolar cell) signals [58, 83, 84], and so will not solely reflect the cone photoreceptor current. Importantly, also, the a-wave trough and initial recovery (termed by some the a-wave “nose”) seen in the dark-adapted response to strong flashes has been shown to be likely to arise from currents flowing in other parts of the photoreceptor [82].

**Fig. 4:**
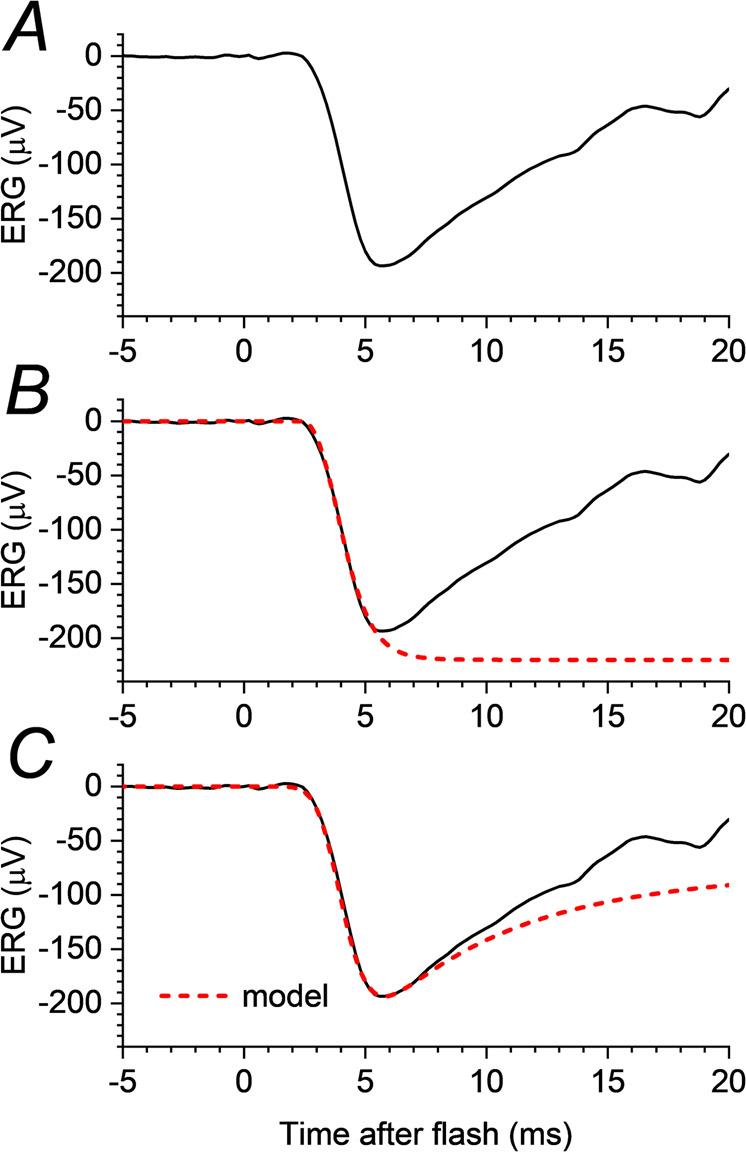
Illustration of mathematical models fitted to rod-driven a-wave response to strong flash. **A** Derived dark-adapted rod-driven response to strong blue flash from a healthy individual; this is the largest amplitude trace from Fig. 3B. This trace is also reproduced in panels **B** and **C**. **B** The red dashed curve shows a fit based on the model of Lamb & Pugh, developed for the outer segment photocurrent. The curve was fitted with the assumption that the photoreceptor response is truncated as a result of the encroaching of post-receptoral signals giving rise to the b-wave (not accounted for in the model), and hence the curve continues downward past the a-wave trough. **C** Red dashed curve shows a fit based on the later model of Robson & Frishman. This model explicitly takes into account current flows in other parts of the photoreceptor layer, showing that the a-wave trough region in the bright-flash ERG is consistent with arising from current flows proximal to the photoreceptor outer segments.

Robson and Frishman have described a more comprehensive mathematical model (taking into account both outer segment as well as photoreceptor axonal currents) that provides a good fit to the rod-driven a-wave elicited by strong flashes, including the a-wave trough and the initial subsequent recovery towards baseline (the nose) prior to the main part of the b-wave [82]. Figure 4 illustrates application of the model to rod-driven responses to a strong flash. Such models could have applicability in photoreceptor diseases, helping elucidate the altered current flows in the outer retina of affected patients.

#### Artificial intelligence algorithms

Artificial intelligence (AI) is being applied to many areas of healthcare showing high levels of accuracy, equivalent to experts. There have been investigations applying AI or machine-learning techniques to electrophysiology data [34, 85–90]. These include studies of ERG data that may have applicability in conditions including glaucoma [88], hydroxychloroquine retinopathy [87], and even autism spectrum disorder [86] and depression [89], as well as studies of VEP data to improve estimation of acuity [90]. A recent study demonstrated that machine learning techniques could be successfully applied to develop automated ERG phenotypic classification of patients with Stargardt disease (arising from variants in the *ABCA4* gene) [85]. This study also used the ERG data to provide a severity score for each *ABCA4* variant (over 1000 different disease-causing variants have been described), which could help give prognostic information for patients based on their genotype. As well as potentially assisting with rapid diagnosis (which could have widespread application given the relative scarcity of ERG expertise), AI techniques could in future yield clinically relevant classifications using subtle features in the waveform data that are undetectable by human experts. AI techniques could also be used to integrate, quantitatively, electrophysiology recordings with imaging and other phenotypic (or genotypic) data to generate predictions of diagnoses, together with levels of certainty.

#### Investigating effects of common genetic variants

Combing electrophysiological analyses with genetic data can be powerful, not just in the field of rare diseases such as *ABCA4*-retinopathy, but also in interrogating effects of common genetic variants on retinal function. ERGs in healthy twins show high heritability [91], indicating that genetic factors are likely to contribute significantly to variance in response parameters in the general population. Large genome-wide association studies have identified many variants associated with common eye diseases. The common variant that is consistently most strongly associated with myopia is near the gene *GJD2* which encodes retinal gap junctions. In the study described earlier [58], a mixed linear model was used, with ERG parameters as outcomes, and allelic dosage at this locus as a predictor, to test the hypothesis that aspects of retinal electrophysiology might associate with this locus. An association was found with some cone-driven ERG parameters, consistent with a possible role for altered cone-driven signals in the pathogenesis of myopia. Studies of this type, particularly if many loci are to be investigated, require large numbers of genotyped individuals with ERG data, and it is anticipated that future investigations in larger cohorts will be informative.

## Concluding remarks

Electrophysiology has provided insights into the physiology and pathophysiology of the human retina and visual pathway for over a century. International standardisation of testing protocols, with regular updates, has been a major advance in bringing about consistency in methods and allowing data generated in laboratories across the world to be meaningfully interpreted. As retinal imaging has advanced tremendously in recent decades, with high resolution cross-sectional images now acquired as a standard in many clinical settings, and single photoreceptors visualised using adaptive optics techniques in research settings, the indications for electrophysiology have evolved, but the tests are far from obsolete. Uniquely, electrophysiology permits direct, quantitative in vivo evaluation of visual pathway function at the level of the retina and visual cortex, and thus provides information not obtainable from imaging. Additionally, such functional testing could potentially detect neuronal dysfunction, for example in diabetic eye disease, prior to changes observable on imaging.

This review is not exhaustive, and many advances have not been covered. With development of newer devices (including portable and multimodal technology), and more refined, including more rapid, testing protocols, combined with novel, AI-assisted analyses, it is likely that tests will become more accessible and continue to yield valuable clinical and scientific information. This will have relevance to common and rare diseases of the eye and visual pathway, and also, given similarities between retinal and brain circuitry, potentially to wider neurological and neuropsychiatric disease [71, 85, 88, 92–94].

## Data Availability

The datasets generated during and/or analysed during the current study are available from the corresponding author on reasonable request.
